# A Fermented Milk Matrix Containing Postbiotics Supports Th1- and Th17-Type Immunity In Vitro and Modulates the Influenza-Specific Vaccination Response In Vivo in Association with Altered Serum Galectin Ratios

**DOI:** 10.3390/vaccines9030254

**Published:** 2021-03-13

**Authors:** Veronica Ayechu-Muruzabal, Ling Xiao, Tjalling Wehkamp, Ingrid van Ark, Elisabeth J. Hoogendoorn, Thea Leusink-Muis, Gert Folkerts, Johan Garssen, Linette E. M. Willemsen, Belinda van’t Land

**Affiliations:** 1 Division of Pharmacology, Utrecht Institute for Pharmaceutical Sciences, Utrecht University, 3584 CG Utrecht, The Netherlands; v.ayechumuruzabal@uu.nl (V.A.-M.); Ling.Xiao@joslin.harvard.edu (L.X.); I.vanArk@uu.nl (I.v.A.); E.J.Hoogendoorn@students.uu.nl (E.J.H.); A.Leusink@uu.nl (T.L.-M.); G.Folkerts@uu.nl (G.F.); Johan.Garssen@danone.com (J.G.); L.E.M.Willemsen@uu.nl (L.E.M.W.); 2 Danone Nutricia Research, 3584 CT Utrecht, The Netherlands; Tjalling.Wehkamp@danone.com; 3 Center for Translational Immunology, The Wilhelmina Children’s Hospital, University Medical Center Utrecht, 3584 EA Utrecht, The Netherlands

**Keywords:** postbiotics, vaccination, influenza, galectins, fermentation, adaptive immunity

## Abstract

During a specific milk fermentation process with *Bifidobacterium breve* C50 and *Streptococcus thermophilus* 065 (Lactofidus^TM^), postbiotics with possible immunomodulatory properties are produced. We investigated the effects of this fermentation product (FP) in vitro using a model that allows crosstalk between intestinal epithelial (IEC) and immune cells. IECs were exposed to FP and αCD3/CD28-activated peripheral blood mononuclear cells after which the mediator secretion was measured. Additionally, using a murine influenza vaccination model, immune development was assessed. Mice were fed an AIN93G diet containing FP or lactose as control. Vaccine-specific immunity was measured as delayed-type hypersensitivity (DTH) and correlated to intestinal and systemic immunomodulation levels. In vitro, exposure to FP enhanced IFNγ, TNFα and IL-17A concentrations. Moreover, IEC-derived galectin-3/galectin-9 and galectin-4/galectin-9 ratios were increased. In vivo, dietary intervention with FP increased vaccine-specific DTH responses as compared to the lactose-receiving group. Although no effects on humoral immunity and vaccine-specific T-cell responses were detected, an enhanced systemic serum galectin-3/galectin-9 and galectin-4/galectin-9 ratio correlated with a shift in RORγ (Th17) mRNA expression over regulatory TGFβ1 in the ileum. This was also positively correlated with the increased DTH response. These results indicate that FP can enhance epithelial galectin-3 and -4 over galectin-9 release, and boost adaptive immunity by promoting Th1- and Th17-type cytokines under inflammatory conditions in vitro. Similar variations in galectin and immune balance were observed in the vaccination model, where FP improved the influenza-specific DTH response.

## 1. Introduction

The development of the mucosal immune system constitutes a crucial stage in early life and its development represents a decisive period for the establishment of a balanced mucosal immune and systemic immune function [1,2]. Diet plays a pivotal role by providing all the necessary nutrients for growth and development of a healthy gut and supporting the establishment of a balanced microbiome and a proper maturation of the immune system. Breastfeeding is considered the gold standard for infant nutrition, and as such, the World Health Organization recommends exclusive breastfeeding during at least the first six months of life, which can be extended up to two years or beyond next to complementary food introduction [3].

Current research is focused on developing alternative nutritional interventions for those infants that are unable to receive enough breastmilk. Although human milk is always the preferred option for infant nutrition, fermented milk-based infant formulas are being developed [4] and studied for their ability to modulate the immune function [5]. Fermented milk-based formulas are obtained by fermentation of a milk matrix with lactic acid-producing bacteria, followed by heat-inactivation of viable bacteria. This fermentation process results in the formation of bioactive components known as postbiotics. Postbiotics are defined as bioactive compounds produced by food-grade microorganisms in a fermentation process (including microbes, cell constituents and metabolites) that in adequate amounts promote health and/or well-being of the host [4,6,7,8]. Postbiotics refer to soluble factors such as enzymes, proteins, polysaccharides, short-chain fatty acids and peptidoglycans, known to promote diverse local as well as systemic effects, among which immunomodulation and anti-inflammation stand out [7].

Specific fermented infant formulas containing postbiotics are commercially available and their possible beneficial effects have been systematically reviewed [9]. The most extensively studied postbiotics are derived from *Lactobacillus*, *Streptococcus* and *Bifidobacterium* strains due to their use as probiotics [6] as well as in regard to the beneficial effects shown in cell-mediated immunity and inflammation [10]. One of the most studied fermented formula is obtained through a unique fermentation process of a milk matrix (Lactofidus™) using two bacterial strains namely *Bifidobacterium breve* C50 and *Streptococcus thermophilus* 065, known to generate bioactive components such as 3′galactosyllactose; a non-digestible oligosaccharide naturally occurring in human milk [11]. Several clinical trials have described improved gut and immune parameters upon dietary intervention with infant formula fermented by *B. breve* C50 and *S. thermophilus* 065, summarized by Salminen et al. [4]. Those clinical trials showed that healthy infants receiving infant formula supplemented with fermentation products from *B. breve* and *S. thermophilus*, had lower severity of acute diarrhea episodes [12], enhanced thymus size [13], lowered fecal calprotectin and increased secretory IgA [14]. Furthermore, systemic effects such as an increased anti-poliovirus IgA response was also seen in infants fed an infant formula supplemented with fermentation products [15]. Additionally, in infants at high risk of atopy who were fed a fermented infant milk formula, fewer cases of positive skin-prick test to cow’s milk were observed [16]. In vitro fermentation products (FP) derived from a fermentation of a milk matrix with *B. breve* C50 and *S. thermophilus* 065 were shown to promote immunomodulatory effects in dendritic cells by increasing IL-10 release [17] as well as stimulating a Th1 immune response in mice [18]. 

In an in vivo influenza vaccination model, specific non-digestible oligosaccharides effectively improved vaccine-specific immune responses by promoting Th1-type immunity [19,20,21,22,23]. Furthermore, in vitro and in vivo combined exposure to non-digestible oligosaccharides and specific bacteria or bacterial fragments (bacterial CpG DNA) has previously been shown to drive regulatory-type Th1 responses, among others, via instruction of epithelial-derived galectin release [24,25,26,27].

Regarding the immunoregulatory capacities of milk-based fermented formula shown in vitro and in vivo, we hypothesize that FP could be able to improve the response to an influenza vaccine in a murine vaccination model by exerting an effect in the gut. Hence, the aim of this study was to investigate the possible immunomodulatory effects of a fermented milk matrix containing possible bioactive fermentation products (FP) produced following the Lactofidus™ fermentation process, and to study its effect in the influenza vaccination model. An established in vitro co-culture model combining human intestinal epithelial as well as immune cells [24,25,28] was used to study the effect of FP on the epithelial cell and immune cells crosstalk. Additionally, a murine influenza vaccination model was used to study the effects of a dietary intervention with FP on vaccination responses. FP was found to modulate the galectin-3 and galectin-4 over galectin-9 balance systemically, which correlated with an increased influenza-specific delayed-type hypersensitivity (DTH) response.

## 2. Materials and Methods 

### 2.1. In Vitro IEC/PBMC Co-Culture Model

#### 2.1.1. Intestinal Epithelial Cell Culture

Human intestinal epithelial cells (IEC), HT-29 cell line (ATCC, HTB-38, Manassas, VA, USA), were cultured in 75 cm^2^ cell culture flasks (Greiner Bio-One, Alphen aan den Rijn, The Netherlands) using Mc Coy 5A medium (Gibco, Invitrogen, Carlsbad, CA, USA) supplemented with 10% heat-inactivated fetal-calf serum (FCS), penicillin (100 U/mL) and streptomycin (100 µg/mL) (Sigma-Aldrich, St. Louis, MO, USA). HT-29 cells were kept in an incubator at 37 °C and 5% CO_2_. Cells were passaged once a week and medium was refreshed every 2–3 days.

#### 2.1.2. Peripheral Blood Mononuclear Cells Isolation

Buffy coats from healthy donor (Sanquin, Amsterdam, The Netherlands) were used to isolate human peripheral blood mononuclear cells (PBMC) by density gradient centrifugation (100 × g, 13 min). After centrifugation, the pellet was washed with PBS supplemented with 2% FCS. The remaining erythrocytes were lysed using red blood cell lysis buffer (4.14 g NH4Cl, 0.5 g KHCO3, 18.6 mg Na2EDTA in 500 mL demi water, sterile filtered, pH = 7.4) for 5 min on ice. The isolated PBMC fraction was resuspended in RPMI 1640 supplemented with 2.5% FCS, penicillin (100 U/mL) and streptomycin (100 µg/mL).

#### 2.1.3. IEC/PBMC Co-Culture Model

One week prior to the experiment, HT-29 cells were diluted five to eight times based on cell surface area and seeded in transwell inserts (12-well, 0.4 µm polyester membrane, Costar Corning Incorporated, NY, USA). When HT-29 reached confluency, they were apically exposed to 0.25–0.5% FP (*w*/*v*). In the basolateral compartment, 2 × 10^6^ cells/mL of αCD3/CD28-activated PBMC (clone CLB-T3/2 and clone CLB-CD28 respectively, both 1:10,000, Sanquin, The Netherlands) were added. After 24 h of incubation (37 °C, 5% CO_2_), the basolateral supernatant was collected and stored at −20 °C for cytokine measurements.

#### 2.1.4. Enzyme-Linked Immunosorbent Assay (ELISA)

The cytokine secretions were analyzed in the basolateral supernatant from IEC/PBMC co-cultures. Commercially available kits were used to determine IFNγ, TNFα, IL-17A, IL-13, TGFβ1 (all from Thermo Fisher scientific, Waltham, MA, USA), IL-10 (U-Cytech, Utrecht, The Netherlands) and galectin-3 (R&D systems, Minneapolis, MN., USA) according to the manufacturer’s protocol. Human galectin-4 and -9 were measured using antibody pairs. In short, high-binding Costar 9018 plates were incubated overnight with 0.75 µg/mL human galectin-4 or -9 affinity purified polyclonal antibody. Non-specific binding was blocked with 1% bovine serum albumin (BSA) in PBS for one hour, after which samples were added and incubated for 2 h at room temperature. After washing, biotinylated galectin-4 or -9 affinity purified polyclonal antibody (0.75 µg/mL) was incubated for 1 h. Then, plates were washed and streptavidin-HRP was incubated for 1 h. After washing, tetramethylbenzidine was used as a substrate to develop the reaction (TMB, Thermo Fisher scientific, Waltham MA, USA), which was stopped with 1 M H_2_SO_4_. Optical density was measured at 450 nm.

### 2.2. In Vivo Influenza Vaccination Model

#### 2.2.1. Animals

Six-week-old C57Bl/6JOlaHsd female mice were purchased from Envigo (Horst, The Netherlands) and housed under conventional conditions with a light/dark cycle of 12 h/12 h (lights on from 7.00 am–7.00 pm) at controlled relative humidity (relative humidity of 50–55%) and temperature (21 ± 2 °C) with access to food and water ad libitum, in the animal facility of Utrecht University. Upon arrival, mice were randomly grouped as three mice per cage in filter-topped makrolon cages (22 cm × 16 cm × 14 cm, floor area 350 cm^2^, Tecnilab BMI, Someren, The Netherlands) with wood-chip bedding (Tecnilab BMI, Someren, The Netherlands); tissues and a plastic shelter were available as cage enrichment at the animal facility. The animals received standard diets for one week until the start of the experiments. The C57Bl/6JOlaHsd female mice were previously used to establish this influenza vaccination model and further studies are also available [19,20,21,22,23,29].

This study was conducted in accordance with institutional guidelines for the care and use of laboratory animals established by the Animal Ethics Committee of Utrecht University, and all animal procedures were approved under the Ethical license of the national competent authority, securing full compliance the European Directive for the use of animals for scientific purposes.

#### 2.2.2. Vaccination Protocol and Dietary Intervention

One week after acclimatization, mice were fed AIN93G diet or the AIN93G diet containing FP or lactose (SNIFF Spezialdiäten GmbH, Soest, Germany) until the end of the experiment by a researcher blinded to the experimental treatments. AIN93G diets were mixed with 0.5% or 2.5% (*w*/*w*) FP. As a control for the amount of lactose present in the fermented milk matrix, the lactose diets were mixed with 0.25% and 1.25% (*w*/*w*), respectively. The percentages of FP and lactose were exchanged against an equal amount (*w*/*w*) of total carbohydrates present in the control diet.

On days 0 and 21, the mice received a subcutaneous vaccination under isoflurane anesthesia using Influvac season 2015/2016 (Abbot Biologicals B.V., Weesp, The Netherlands). The mice (*n* = 9 per experimental group) received a total volume of 100 µL containing 90 µg/mL hemagglutinin from three strains of the influenza virus. A negative control group, referred as sham (*n* = 3), received injections with 100 µL PBS. Delayed-type hypersensitivity reactions were induced 9 days after booster vaccination by intradermal injection of 20 µL Influvac into the ear pinnae of the right ear. As basal line, 20 µL PBS was injected in the left ear pinnae. Ear thickness was measured in duplicate before intradermal challenge and 24 h thereafter using a digital micrometer (Mitutoyo, Veenendaal, The Netherlands). The antigen-specific delayed-type hypersensitivity (DTH) responses were calculated using the following formula: DTH = Right ear thickness (@24 h–@0 h)–Left ear thickness (@24 h–@0 h). After measuring the ear thickness, mice were anesthetized and sacrificed. Then, the ears were punctured and the weight of the ear puncture was measured.

#### 2.2.3. Vaccine-Specific Immunoglobulins and Galectins in Serum

At the end of the experiment, mice were anesthetized and blood was collected by retro-orbital bleeding of the eye. Blood samples were centrifuged (14,000 rpm, 10 min) and serum was stored at −80 °C until analysis of vaccine-specific antibodies by means of ELISA. Vaccine-specific antibody titers were measured as described previously [19]. Briefly, 96-well high-binding plates (Costar Corning Incorporated, NY, USA) were coated with 1:100 diluted Influvac in PBS. As blocking reagent, 2% BSA in PBS was used. Serial dilutions of pooled serum containing vaccine-specific antibodies was done and used for standard curve calculations. Biotinylated anti-IgG1 and anti-IgG2a antibodies (Becton Dickinson, Heerhugowaard, The Netherlands) were diluted 1:100 and incubated for 1 h, after which streptavidin-HRP (Sanquin, Amsterdam, The Netherlands) was added. Optical density was measured at 490 nm with a microplate reader (Bio-Rad, Veenendaal, The Netherlands). Mouse serum galectin-4 and -9 (both from R&D systems) were measured by means of ELISA according to the protocol described in Section 2.1.4. for human galectin-4 and -9. Purified mouse anti-goat antibodies (0.2 mg/mL for galectin-4 and -9), recombinant mouse cytokines and biotinylated goat anti-mouse antibodies (0.2 mg/mL for galectin-4 and -9) were purchased from R&D systems. Non-specific binding was blocked using PBS supplemented with 5% goat serum (Dako, Heverlee, Belgium). Mouse galectin-3 concentrations were measured according to manufacturer’s protocol (R&D systems).

#### 2.2.4. Cell Isolation from Tissues

Lymphocytes were isolated from the spleens and mesenteric lymph-nodes (MLN) of mice sacrificed after the ear thickness measure, 24 h after challenge. Single-cell splenocyte suspensions were obtained by crushing the spleen through a 70 µm nylon cell strainer using a syringe. The splenocyte cell suspensions were incubated with a lysis buffer (8.3 g NH4Cl, 1 g KHCO3 and 37.2 mg EDTA dissolved in 1 L demi water, filter-sterilized) for 4 min on ice to remove the red blood cells. Cell suspensions were resuspended in RPMI 1640 medium (Lonza, Basel, Switzerland) supplemented with 10% heat-inactivated FCS, 100 U/mL penicillin, 100 µg/mL streptomycin and 20 µM β-mercapto-ethanol (Thermo Fisher). 

#### 2.2.5. Flow Cytometry of Immune Cells

Spleen and MLN single cell suspensions (0.5–1 × 10^6^ cells/well) were incubated with anti-mouse CD16/CD32 (Mouse BD Fc Block, BD Biosciences, San Jose, CA, USA) in PBS supplemented with 1% BSA and 5% FCS for 15 min on ice to block non-specific binding sites. Subsequently, cells were incubated for 30 min with the following antibodies: CD4-PerCP Cy5.5, CCR6-PE (both from Biolegend, San Diego, CA, USA) CD8a-PECy7, CD69-PE, CD25-Alexa Fluor 488, CD3-PerCP Cy5.5, CD27-PE, CD19-APC and B220-FITC (all from Thermo Fisher). For intracellular staining, cells were first fixated and permeabilized with Foxp3 Staining buffer set (Thermo Fisher) according to manufacturer’s protocol, followed by incubation with Foxp3-PECy7 (Thermo Fisher), RORγT-Alexa Fluor 647 (BD) or Tbet-eFluor 660 (Biolegend). Dead cells were excluded using Fixable Viability Dye eFluor^®^ 780 (Thermo Fisher). Stained cells were measured by FACS Canto II (BD Biosciences) and analyzed using Flowlogic software version 7 (Inivai Technologies, Mentone, VIC, Australia). 

#### 2.2.6. Generation of Bone Marrow-Derived Dendritic Cells (BMDC)

Naïve mice (donor mice) were sacrificed on day 24 and bone marrow cells were isolated from the femur as previously described [23,30,31]. Bone marrow cells were resuspended in RPMI 1640 supplemented with 10% FCS, penicillin (100 U/mL) and streptomycin (100 µg/mL). Bone marrow cell suspensions (0.5 × 10^6^ cell/mL) were cultured in the presence of 20 ng/mL recombinant mouse granulocyte macrophage colony-stimulating factor (GM-CSF) (Prospec, Rehovot, Israel) in a petri dishes (Corning). On day 3, medium was refreshed, and on day 6, bone marrow derived-dendritic cells (BMDC) were harvested. Immature BMDC were then loaded with the Influvac vaccine (0.9 µg/mL) and incubated for 24 h (37 °C, 5% CO_2_). 

#### 2.2.7. Ex Vivo Re-Stimulation Assay

Spleens were aseptically removed and single cell suspensions were obtained as described in Section 2.2.4. Splenocytes (5 × 10^6^) were co-cultured with BMDC (5 × 10^5^) either or not loaded with Influvac in U-bottom 96-well plates at 37 °C, 5% CO_2_. After 5 days of incubation, supernatants were collected and stored at −20 °C for cytokine analysis. IFNγ, IL-13 (both from R&D) were measured by means of ELISA according to the protocol described in Section 2.1.4. for galectin-4 and -9. Purified rat anti-mouse antibodies (1 µg/mL for IFNγ and 2 µg/mL for IL-13), recombinant mouse cytokines and biotinylated rat anti-mouse antibodies (1 µg/mL for IFNγ and 400 ng/mL for IL-13) were purchased from BD Biosciences. TNFα (Biolegend), IL-10 and IL-17A concentrations (both from Thermo fisher) were measured by ELISA according to manufacturer’s protocol.

#### 2.2.8. qPCR Analysis of Gene Expression

Ileum and colon samples from mice sacrificed after DTH measurement were collected in RNA later (Invitrogen) and stored in −80 °C until mRNA isolation. Tissues were homogenized and RNA was isolated using a NucleoSpin^®^ RNA Plus kit (Macherey-Nagel, Düren, Germany) in combination with DNAse (Qiagen, Hilden, Germany) to remove contaminating DNA. Complementary DNA (cDNA) was synthesized using an iScript™ cDNA synthesis kit (Bio-Rad) according to the manufacturer’s protocol. Quantitative analysis was performed on a CFX96 real-time PCR detection system with the use of IQ™ SYBR^®^ Green Supermix (both from Bio-Rad). Commercially available primers for TGFβ1, TGFβ3, RORγ, Foxp3, TNFα, IL-10, Tbx21, galectin-3, -4 and -9, were obtained and GAPDH and PPIP5K1 (all from Qiagen) were used as reference genes. Relative mRNA expression was calculated as 100 × 2^Ct reference − Ct gene of interest^ [32]. Additionally, a custom-designed primer (Table 1) was used for, TNFα (Biolegio, Nijmegen, The Netherlands), previously validated [23].

### 2.3. Statistical Analysis

All statistical analyses were done using GraphPad Prism software (San Diego, CA, USA). Data were transformed if they did not fit normal distribution prior to ANOVA analysis. IEC/PBMC co-culture datasets were analyzed using one-way ANOVA followed by Bonferroni’s post-hoc test. The sample size of the in vivo vaccination study was calculated based on the DTH results from previous studies. The in vivo datasets from the vaccination model were analyzed using a one-way ANOVA followed by Bonferroni’s test with selected pairs. Probability values of *p* < 0.05 were considered significant. 

## 3. Results

### 3.1. Exposure of IEC to FP Enhances Th1- and Th17-Type Cytokines Iin the IEC/PBMC Co-Culture

**Figure 1:**
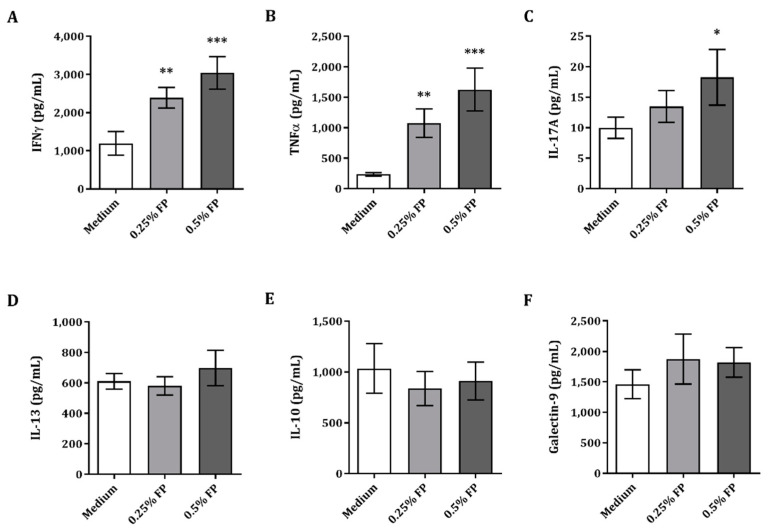
Cytokine release in IEC/PBMC co-culture. IECs were apically exposed to 0.25% or 0.5% FP and basolaterally to αCD3/CD28-activated PBMC. IFNγ (**A**), TNFα (**B**), IL-17A (**C**) IL-13 (**D**), IL-10 (**E**) and galectin-9 (**F**) concentrations were measured in the basolateral supernatant after 24 h co-culture. Data are represented as mean ± SEM of 5–6 independent PBMC donors. Significant differences are shown as * *p* < 0.05, ** *p* < 0.01, *** *p* < 0.001.

A model to study the crosstalk between IEC and innate as well as adaptive immune cells was used to investigate the immunomodulatory effects of FP. Therefore, IECs were apically exposed to FP and basolaterally to αCD3/CD28-activated PBMC for 24 h, after which cytokines were analyzed. Exposure of IEC to activated PBMC and 0.25% or 0.5% FP resulted in significantly increased Th1-type IFNγ and TNFα concentrations as compared to medium (Figure 1A,B). Th17-type IL-17A concentrations were significantly upregulated upon exposure to 0.5% FP, as compared to medium (Figure 1C). Meanwhile, Th2-type IL-13 and regulatory-type IL-10 and galectin-9 concentrations were not affected upon exposure to FP in the IEC/PBMC model (Figure 1D–F). The secretion of Th1- and Th17-type cytokines was promoted in the IEC/PBMC model upon exposure to FP.

### 3.2. IEC-Derived Galectin-3, -4 and-9 after IEC/PBMC Co-Culture

**Figure 2:**
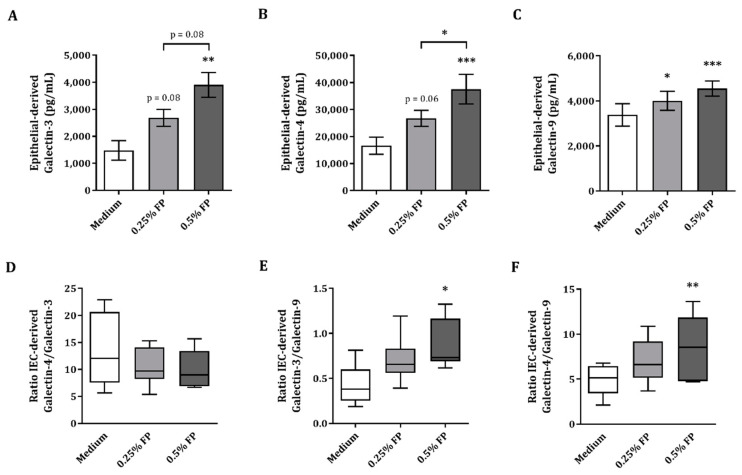
Epithelial-derived galectin release after IEC/PBMC co-culture. IECs were apically exposed to 0.25% or 0.5% FP and basolaterally to αCD3/CD28-activated PBMC for 24 h. After IEC/PBMC co-culture, IECs were washed and incubated with fresh medium for additional 24 h to measure epithelial-derived galectin-3 (**A**), -4 (**B**) and -9 (**C**) concentrations. Additionally, the ratios between epithelial-derived galectins are shown. Shown are the galectin-4 and -3 ratio (**D**), the galectin-3 and -9 ratio (**E**) and the galectin-4 and -9 ratio (**F**). Data are represented as mean ± SEM of 5–6 independent PBMC donors. Significant differences are shown as * *p* < 0.05, ** *p* < 0.01, *** *p* < 0.001.

In order to analyze the epithelial cell responsiveness, after the IEC/PBMC co-culture, the IECs were washed and kept in incubation with fresh medium for an additional 24 h, after which IEC-derived mediator release was measured. Due to their involvement in the regulation of many immune processes, IEC-derived galectin concentrations were studied in the basolateral compartment. Exposure to 0.5% FP resulted in significantly increased IEC-derived galectin-3, -4 and -9 (Figure 2A–C) as compared to control or 0.25% FP. IEC-derived galectin-9 was significantly increased upon exposure to 0.25% FP (Figure 2C). Meanwhile, a tendency towards increased epithelial-derived galectin-3 and -4 (*p* = 0.08 and *p* = 0.06 respectively, Figure 2A,B) was observed upon exposure to 0.25% FP, compared to medium. Another regulatory mediator known to be produced by epithelial cells is TGFβ1, but FP did not enhance TGFβ1 concentrations above medium background levels (data not shown).

As the rise in galectin-3 and -4 release upon 0.5% FP exposure appeared greater than the rise in galectin-9 release, the ratios between IEC-derived galectins was calculated to illustrate the balance between these inflammatory and regulatory galectins. No significant effect was found in the ratio of galectin-4 over galectin-3 (Figure 2D), whereas the ratio of galectin-3 over galectin-9 (Figure 2E) and galectin-4 over galectin-9 (Figure 2F) showed a significant increase in the 0.5% FP conditions. Hence, exposure of IEC to FP in the IEC/PBMC model resulted in significantly increased IEC-derived galectin-3, -4 and -9, while the ratio of galectin-4 over galectin-9 as well as the ratio of galectin-3 over galectin-9 significantly increased upon exposure to 0.5% FP.

In light of the immunomodulatory profile shown by FP in the IEC/PBMC model, further studies were done to determine the capacity of FP on the improvement of influenza vaccination responses in vivo.

### 3.3. Dietary Intervention with FP Improves the Vaccine-Specific DTH Response

**Figure 3:**
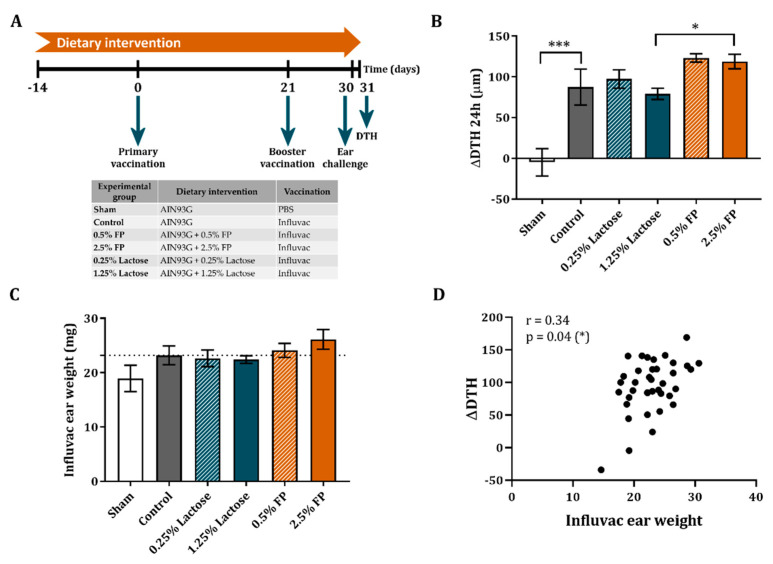
The effect of a dietary intervention with FP on the vaccine-specific DTH response and ear weight in an influenza vaccination model. The study design is shown (**A**). The increase in DTH response after ear challenge (**B**) and the Influvac ear weight (**C**) 24 h after challenge are shown. The spearman correlation between the ΔDTH response and the ear weight is shown (**D**). Data are represented as mean ± SEM of sham (*n* = 3) and vaccinated mice (*n* = 9). Significant differences are show as * *p* < 0.05, *** *p* < 0.001.

On day 30, the C57BL/6OlaHsd mice received a subcutaneous injection with the vaccine in the ear pinnae, after which, on day 31, the ear swelling was measured as DTH to determine the T-cell-dependent cellular response to vaccination. A significant increase in the influenza-specific DTH response was seen in all vaccinated mice as compared to the non-vaccinated sham mice (Figure 3B). The DTH response did not differ between the vaccinated mice receiving lactose diet as compared to vaccinated mice receiving control diet (Figure 3B). Although 0.5% FP did increase the DTH reaction compared to its appropriate lactose control, it did not reach the level of significance. However, increasing the dose to 2.5% FP significantly enhanced the DTH response compared to its appropriate lactose control group (Figure 3B).

Although no significant effect was observed in the weight of the Influvac injected ears, a similar pattern compared to the ΔDTH response was observed (Figure 3C). In addition, the differences detected in the weight of the Influvac injected ears significantly correlated response with the ΔDTH (r = 0.34, *p* = 0.04) (Figure 3D). Due to the significantly increased vaccine-specific DTH response observed in the mice receiving 2.5% FP diet, further analyses were done in this group and its respective lactose control group.

### 3.4. Influvac-Specific Igg1 and Igg2a in Serum and Ex Vivo Cytokine Secretion

**Figure 4:**
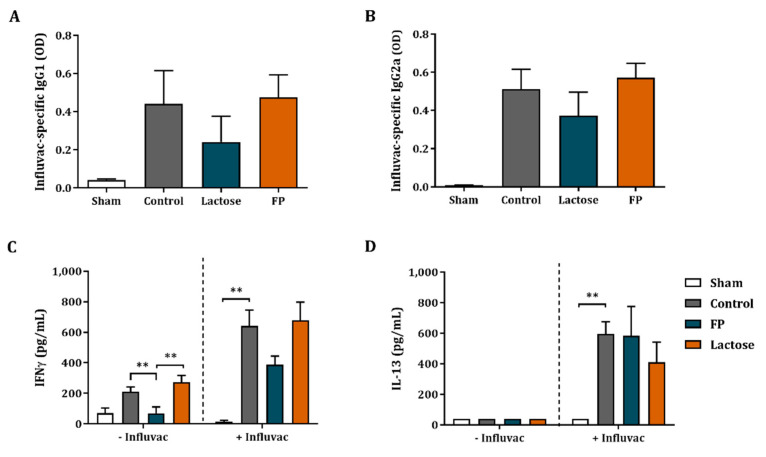
Serum immunoglobulins and cytokine production after ex vivo re-stimulation with influenza-loaded BMDCs. Influvac-specific IgG1 (**A**) and IgG2a (**B**) concentrations were measured in the serum. Additionally, BMDCs were obtained from donor mice and loaded with or without 0.9 µg/mL Influvac for 24 h. On day 31, BMDCs were co-cultured with fresh spleen cell suspensions from sham as well as vaccinated mice for 5 days, after which the supernatants of the co-culture were collected and the cytokine secretion was analyzed. IFNγ (**C**) and IL-13 (**D**) concentrations are shown. Data are represented as mean ± SEM of sham (*n* = 3) and vaccinated mice (*n* = 9). Significant differences are show as, ** *p* < 0.01.

In order to measure the humoral responsiveness to the vaccine and the impact of the dietary intervention with FP, the serum of the mice was collected and vaccine-specific IgG1 and IgG2a were measured. There was an increase in IgG1 and IgG2a levels in vaccinated mice compared to non-vaccinated sham mice (Figure 4A,B). However, vaccine-specific IgG1 and IgG2a levels were not affected by the dietary interventions with FP or lactose (Figure 4A,B).

In order to investigate the effect of FP in the systemic vaccination response both T- and B-cell subsets of the spleen and MLN were analyzed by flow cytometry. Regulatory T-cells were identified as CD4^+^CD25^+^Foxp3^+^, RORγ positive cells were identified as CD4^+^CCR6^+^RORγ^+^ and, lastly, activated Th1 type cells were identified as CD4^+^CD69^+^Tbet^+^. Activated B-cells were identified as CD3^-^CD19^+^B220^+^CD27^+^ and activated CD8^+^ T-cells as CD8^+^CD69^+^.

In the spleen, the frequency of regulatory T-cells was significantly increased, and the frequency of activated Th1-type cells was decreased in vaccinated mice as compared to non-vaccinated mice; however, this was not affected by the dietary intervention (Figure S1). Dietary intervention with FP did also not have an effect in the T- and B-cell frequencies of the spleen, even though in the lactose control group the frequency of activated B- and CD8 T-cells was increased as compared to the control diet (Figure S1). In the MLN, no significant differences were found in either T- or B-cell populations of vaccinated mice as compared to non-vaccinated mice. Dietary intervention with FP also did not affect the T- and B-cell frequencies in the MLNs (Figure S1). The frequency of Th1 positive T-cells in the MLNs was significantly increased in the mice receiving lactose as compared to control and FP (Figure S1).

Using an ex vivo re-stimulation model, vaccine-specific T-cell responses were investigated. Splenocyte cell suspensions were co-cultured with BMDC either loaded or not with Influvac for 5 days. After co-culture, the cytokine concentrations were analyzed. Co-culture of splenocytes with non-loaded BMDC resulted in a relatively small non-specific background increase of IFNγ concentrations, while IFNγ was significantly increased when using Influvac loaded BMDC (Figure 4C). Similarly, increased IL-13 concentrations were found in co-cultures of splenocytes from vaccinated mice as compare to non-vaccinated mice in co-culture with Influvac-loaded BMDC (Figure 4D). Dietary intervention with lactose or FP did not further increase IFNγ and IL-13 concentrations (Figure 4C,D). TNFα, IL-17A and IL-10 concentrations were under detection limit.

Dietary intervention with FP did not have an effect the Influvac-specific IgG1 and IgG2a levels or the frequency of B-cell populations in spleen of MLNs. Influenza-specific ex vivo re-stimulation induced IFNγ secretion was not altered in the FP group.

### 3.5. Shift in Th17/T-Regulatory Mrna Expression in Ileum

**Figure 5:**
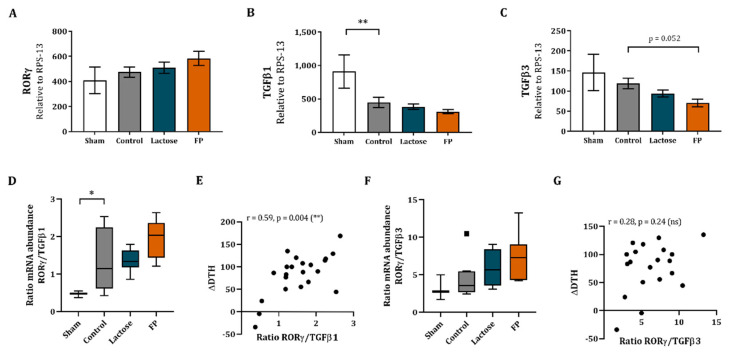
Relative mRNA expression in ileum and correlation with ΔDTH. Relative mRNA expression of RORγ (**A**), TGFβ1 (**B**) and TGFβ3 (**C**) were measured in the ileum using RT-qPCR. RORγ/TGFβ ratios were calculated to represent Th17/regulatory balance in ileum. The ratio of RORγ over TGFβ1 mRNA abundance (**D**) and RORγ over TGFβ3 (**F**) are shown. Additionally, Spearman correlations of ΔDTH and RORγ over TGFβ1 ratio (**E**) as well as correlations of ΔDTH and RORγ over TGFβ3 ratio (**G**) are shown. Data are represented as mean ± SEM of sham (*n* = 3) and vaccinated mice (*n* = 9). Significant differences are show as * *p* < 0.05, ** *p* < 0.01.

The effect of dietary intervention with FP was also assessed locally in the intestine. Therefore, ileal as well as colonic sections were subjected to qPCR analysis, which obtained detectable levels of RORγ, TGFβ1 and TGFβ3. Meanwhile, Foxp3, TNFα, IL-10 and Tbx21 were below detection limits. Although no effects were observed in the relative mRNA abundance of RORγ (Figure 5A), the TGFβ1 relative mRNA abundance was decreased in the ileum of vaccinated mice as compared to non-vaccinated mice (Figure 5B). Even though in the FP diet group an increasing pattern of RORγ mRNA expression and a decreasing pattern of TGFβ1 expression was shown, this did not reach statistical significance (Figure 5A,B). The TGFβ3 mRNA expression did tend to decrease in the ileum of the mice receiving FP diet as compared to control (Figure 5C).

Additionally, to represent the Th17 immune versus regulatory balance in the ileum, the ratio of Th17 marker RORγ and regulatory marker TGFβ was calculated (RORγ/TGFβ1 and RORγ/TGFβ3). The RORγ/TGFβ1 ratio was significantly increased in vaccinated mice as compared to non-vaccinated mice (Figure 5D). No significant effect was found in RORγ/TGFβ1 ratio between control and FP groups (Figure 5D), although the FP showed a shift towards RORγ over TGFβ1 compared to the control and lactose groups. In addition, the correlation between RORγ/TGFβ1 ratio and ΔDTH response was studied, which showed a significant positive correlation (r = 0.59, *p* = 0.004, Figure 5E). No effect was found in the RORγ/TGFβ3 ratio (Figure 5F) and no correlation was found between the RORγ/TGFβ3 ratio and ΔDTH (r = 0.28, *p* = 0.24, Figure 5G).

In the colon, the relative mRNA abundance of RORγ in vaccinated mice was decreased as compared to non-vaccinated mice (Figure S2). No effects were found in the relative mRNA abundance of galectin-3, galectin-4, galectin-9, TGFβ1 or TGFβ3 in vaccinated mice as compared to non-vaccinated mice (Figure S2). TGFβ3 mRNA expression was significantly increased by FP and lactose, as compared to control. The TGFβ1, RORγ and galectin-3, -4, and -9 mRNA abundance was not affected upon dietary intervention with FP (Figure S2).

### 3.6. Galectin-3, -4 and -9 Mrna Expression in Ileum and Concentrations in Serum

Besides the immune markers, the impact of the dietary intervention by FP was studied locally on the gene expression of galectins in the ileum and colon. No effect of the vaccination or the FP diet was found in the relative mRNA abundance of galectin-3, -4 and -9 (Figure S3).

**Figure 6:**
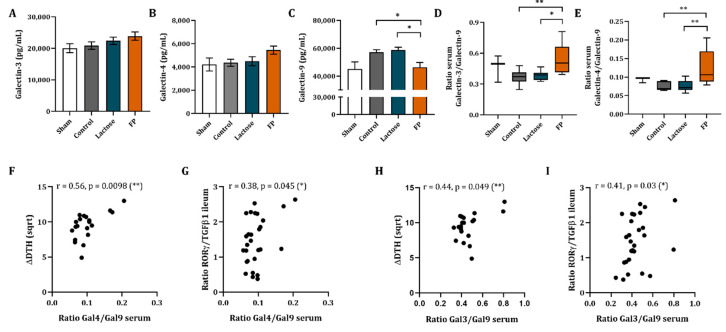
Galectin-3, -4 and -9 concentrations in serum. Serum galectin-3 (**A**), galectin-4 (**B**) and galectin-9 (**C**) concentrations were measured. The galectin-4/galectin-9 ratio (**D**) and galectin-3/galectin-9 ratio (**E**) are shown. Serum galectin ratios were correlated to ΔDTH and RORγ/TGFβ1 ratio in the ileum. Spearman correlation of galectin-4/galectin-9 ratio and ΔDTH is shown (**F**). Galectin-4/galectin-9 ratio was also correlated with RORγ/TGFβ1 ratio using a Pearson correlation (**G**). Additionally, correlations of galectin-3/galectin-9 ratio and ΔDTH (**H**) as well as to the RORγ/TGFβ1 ratio (**I**) were calculated using a Pearson and Spearman correlation, respectively. Data are represented as mean ± SEM of sham (*n* = 3) and vaccinated mice (*n* = 9). Significant differences are show as * *p* < 0.05, ** *p* < 0.01.

Galectin concentrations were also measured in the serum of the mice. No significant differences were observed in galectin-3, -4 and -9 concentrations in the serum of vaccinated mice as opposed to non-vaccinated mice (Figure 6A–C). Galectin-3 and -4 concentrations were not affected upon dietary intervention with FP or lactose as compared to control group. However, for the group fed the FP diet, an increasing pattern was observed (Figure 6A,B), while galectin-9 concentrations in serum were reduced as compared to both the control and lactose group (Figure 6C). As a result, the galectin-4/galectin-9 as well as the galectin-3/galectin-9 ratio were significantly increased (Figure 6D,E, respectively) in the FP group as compared to the control and lactose group. The galectin-4/galectin-3 ratio was not affected (Figure S4).

In order to connect the effects observed, such as the increase in DTH as well as of RORγ/TGFβ1 ratio in the intestine, with the serum galectin concentrations, the galectin-4/galectin-9 and galectin-3/galectin-9 ratio were correlated to RORγ/TGFβ1 ratio in the ileum as well as to ΔDTH. The serum galectin-4 over galectin-9 ratio showed a positive correlation to ΔDTH (r = 0.56, *p* = 0.0098, Figure 6F) and to the ratio of RORγ/TGFβ1 mRNA expression in the ileum (r = 0.38, *p* = 0.045, Figure 6G). Furthermore, the serum galectin-3/galectin-9 ratio was found to be significantly correlated to ΔDTH (r = 0.44, *p* = 0.049, Figure 6H) as well as to the ratio of RORγ/TGFβ1 mRNA expression in ileum (r = 0.41, *p* = 0.03, Figure 6I). Meanwhile, RORγ/TGFβ3 ratio did not show a significant correlation (Figure S4) with serum galectin-4/galectin-9 ratio (r = 0.25, *p* = 0.2) or galectin-3/galectin-9 ratio (r = 0.3, *p* = 0.12).

Dietary intervention with FP significantly reduced galectin-9 concentrations in the serum, which contributed to significantly increased galectin-4 over galectin-9 as well as galectin-3 over galectin-9 ratios. This shift in serum galectin-4/galectin-9 and galectin-3/galectin-9 correlated with an increase in DTH as well as to an increase in the ratio of RORγ/TGFβ1 mRNA expression in the ileum.

## 4. Discussion

Postbiotics are known to improve immune as well as gut parameters in healthy and pre-term infants [4]. An in vitro co-culture model developed to study the crosstalk of IEC and immune cells was used to investigate the immunomodulatory capacity of FP derived from the fermentation of a milk matrix with *Bifidobacterium breve* C50 and *Streptococcus thermophilus* 065 (Lactofidus™). Studies using the same model showed the immunomodulatory capacity of non-digestible oligosaccharides in association with CpG ODN, a TLR9 agonist, under inflammatory conditions [21,22,28]. In the current study, apical exposure of IEC to FP, and basolaterally to αCD3/CD28-activated PBMC, resulted in significantly increased Th1-type IFNγ and TNFα, as well as Th17-type IL-17A concentrations. Moreover, Th2-type IL-13, regulatory-type IL-10 and galectin-9 were not affected, which indicates that FP can boost the adaptive immunity by promoting Th1- and Th17-type cytokine release in this model. Unlike the studies with specific non-digestible oligosaccharides, namely a 9:1 mixture of short-chain galacto- and fructo-oligosaccharides (GF) [24,28] or 2′-fucosyllactose (2′-FL) [22] and TLR9 agonist CpG ODN, FP did not boost IL-10 nor lowered IL-13, which evidences selective FP immunomodulatory properties. However, as opposed to non-digestible oligosaccharides in association with CpG ODN, FP promoted a strong Th1- and Th17-type response with less regulatory component already in the absence of CpG ODN, again emphasizing the relevance of the particular properties of FP.

IEC-derived galectin-9 has been identified as a key factor contributing to immunomodulation by previous studies using the IEC/PBMC model [24,25,28]. For the purpose of this study, we used the HT-29 cell line as a model for IEC. HT-29 have previously been shown to differentially respond to diverse microbial or immune triggers mimicking responses in human intestinal biopsies [33,34]. The HT-29 also were shown to have a similar immunomodulatory effect compared to the differentiated T84 epithelial cell model when co-cultured in transwells with PBMC. Similar to HT-29, also the T84 cell line expressed and secreted galectin-9 upon apical exposure to TLR9 agonist CpG ODN, resulting in increased IFNγ secretion by the underlying PBMC [35]. The involvement of IEC-derived and/or systemic galectin-9 as an immunomodulatory factor has also been substantiated in dietary intervention studies done in food allergy prevention models as well as in human infants [26,27]. The confirmation of findings in the HT29/PBMC co-culture model in (pre)clinical settings further validates the choice of HT-29 as a model for intestinal epithelial cells to study the crosstalk between IEC and immune cells.

Besides galectin-9, the association of IEC-derived galectin-3 and -4 in promoting immunomodulatory effects in the IEC/PBMC model was already reported upon exposure to non-digestible oligosaccharides and CpG ODN [22]. Galectins are carbohydrate-binding proteins that function to modulate innate and adaptive immune responses. Secreted by epithelial as well as immune cells, galectins are key players in inflammatory and regulatory immune processes [36]. Galectin-3 as well as -4 were shown to have anti-inflammatory as well as pro-inflammatory activities in diverse immune processes [37,38]. Besides its role in the stabilization of lipid rafts, apical protein trafficking and cell adhesion [38], galectin-4 was shown to exacerbate intestinal inflammation by stimulating CD4+ T-cells to produce IL-6 in a murine colitis model [39]. Contrarily, galectin-4 has also been described as an anti-inflammatory agent by selectively modulating T-cell responses in an experimental colitis model [40]. Similarly, galectin-3 also showed anti-inflammatory properties by contribution to ameliorate mucosal inflammation in a murine colitis model [41]. In this regard, galectin-9 has been shown to regulate inflammatory responses and collaborates with TGFβ to instruct regulatory T-cell development [42,43]. In the current study, exposure to 0.5% FP resulted in significantly increased IEC-derived galectin-3, -4 as well as -9, which indicates that not only galectin-9, but also galectin-3 and -4 might be involved in the immunomodulatory effects promoted by FP. However, upon exposure to FP, the ratio of IEC-derived galectin-3 or galectin-4 over galectin-9 were significantly increased, suggesting a more immunostimulatory over regulatory profile induced by FP.

Due to the ability of FP in boosting the adaptive immunity by promoting Th17- and Th1-type cytokines in the IEC/PBMC model, the capacity of a diet containing FP in improving a vaccination immune response was studied. Thereby, an established in vivo influenza vaccination model was used [19] in which mice received a dietary intervention with FP or lactose as a control. Dietary intervention with non-digestible oligosaccharides can modulate the vaccine-specific DTH response, a Th1-related parameter [20,21,22,23,29]. In line with these studies, here, we show that the vaccine-specific DTH was increased, although not significantly, upon dietary intervention with 0.5% FP compared to the lactose control diet. However, increasing the dose of the dietary intervention to 2.5% FP resulted in a significant increase in the DTH response. The increase in vaccine-specific DTH in vivo and the increase in Th1 and Th17-type cytokines as well as the increased IEC-derived galectin-3 or galectin-4 over galectin-9 ratio seen in the IEC/PBMC model in vitro highlight the ability of FP to support Th1- and Th17-type immunity possibly in association with the modulation of galectin expression.

Despite the increase in DTH observed in mice receiving the FP-containing diet, the vaccine-specific IgG1 and IgG2a levels were not affected by the dietary intervention with FP. Previous studies described similar effects in mice receiving a GF supplemented diet [19]. Contrarily, significantly increased IgG1 and IgG2a levels were found in mice receiving a 2′-FL supplemented diet [23]. This suggests that FP supplementation might affect the T-cell rather than B-cell immune responses, emphasizing the selective mechanisms derived from different dietary interventions.

Changes in the phenotype of T- and B-cells were studied in the spleen and MLNs of the mice and Influvac re-stimulation was performed ex vivo. The increase in DTH did not translate into an increase in the percentage of Th1 or Th17 type T-cells in the spleen or MLN, measured as CD69^+^Tbet^+^ or CCR6^+^RORγ^+^, nor enhanced Influvac-specific IFNγ release, suggesting that such an effect is obtained through distinct mechanisms.

Regulatory T-cells as well as Th17 cells are found in intestinal mucosal immune responses and are known to protect the host from exaggerated effector T-cell responses. Th17 cells were described to have a critical role in host defense and vaccine-induced memory immune responses, by promoting the recruitment of Th1-type cells through the upregulation of chemokines, among other processes [44,45]. TGFβ is a key mediator involved in regulating the differentiation of naïve T-cells into regulatory as well as Th17-type. Thus, the Th17/Treg balance is key in maintaining gut immune homeostasis [46,47,48]. In order to study the effects of the dietary intervention with FP in the Th17/Treg balance in the intestine, RORγ and TGFβ1 mRNA expression of the ileum and colon were studied. Dietary intervention with FP was found to enhance the balance of RORγ over TGFβ1, which was calculated as a reflection of the Th17/Treg balance. This ratio was enhanced in the intestine upon systemic vaccination and correlated positively with the DTH response. This indicates that the modulation of the intestinal immune system by means of a dietary intervention with FP might be able to affect the systemic vaccine-specific immune response.

In addition to TGFβ1, mRNA expression of TGFβ3 was measured. Dietary intervention with 2′-FL significantly increased mRNA expression of TGFβ3 in a murine influenza vaccination model [23]. In the current study, as opposed to 2′-FL, relative mRNA abundance of TGFβ3 tended to decrease upon dietary intervention with FP as compared to control, showing a similar trend as seen for TGFβ1 mRNA expression. However, as opposed to the RORγ/TGFβ1 ratio, the RORγ/TGFβ3 ratio and ΔDTH were not correlated. Therefore, in addition to these immune markers, the role for galectins in the immunomodulatory effect of the FP diet were further studied.

Circulating galectins are being considered as relevant biomarkers for supporting the diagnosis of several chronic disorders [49]; even in response to viral infections such as influenza, plasma galectin-9 levels were found to be a relevant biomarker for disease prognosis [50]. In order to determine the relevance of circulating galectins in our model and link them to the epithelial-derived galectin concentrations seen in vitro, serum concentrations of galectin-3, -4 and -9 were studied. Dietary intervention with FP showed decreased galectin-9 concentrations, while no effects were found in galectin-3 and -4 concentrations. Indeed, galectin-3, -4 and -9 could also be measured in the intestine and serum of the mice in the vaccination model. Even though different responses were observed regarding the modulation of galectin levels in the murine vaccination model as compared to the in vitro IEC/PBMC co-culture, these might derive from the specific conditions mimicked in the models. While the IEC/PBMC co-culture represents a generic inflammation in vitro, the vaccination model focuses on antigen-specific immune responses in vivo, where more complex immune processes are studied. In spite of the distinct individual galectin concentrations observed in the serum of the mice, the galectin-4/galectin-9 ratio as well as the galectin-3/galectin-9 ratio were significantly increased upon exposure to FP in the in vivo model, similar to the IEC/PBMC co-culture model. This points towards a similar role of these types of galectins in orchestrating the immune activation and highlights the translational value of the in vitro co-culture model, which includes both IEC as well as immune cells, when studying effects on immune activation. Thus, validating the relevance of the results observed in less complex in vitro models as compared to in vivo models. Moreover, the use of in vitro models could contribute to evaluating the effects of bioactive components in order to select the most promising intervention and condition to be confirmed in animal studies, thereby contributing to the reduced use of animals.

Furthermore, the serum galectin-4/galectin-9 ratio as well as the galectin-3/galectin-9 ratio appeared to be correlated to the increased vaccine-specific DTH. This supports the idea that the circulating galectin balance was affected by the FP diet, which as a consequence might have an effect in the vaccine-specific immune response as measured by means of the DTH response. Little is known about the role of galectins in vaccination. However, galectins have shown dual-regulatory capacities in the promotion or inhibition of viral infections depending on the surrounding conditions and localization [51,52]. This study reveals a potential involvement of systemic galectins in the improvement of vaccine immune responses. Further research is needed to study the contribution of specific subtypes of galectins in this regard.

Altogether, a Th17- and Th1-type immunomodulatory capacity of FP was shown in the IEC/PBMC model associated with increased epithelial-derived galectin-4 and galectin-3 over galectin-9. Although there was no significant difference in serum galectin-3 and -4 upon dietary intervention with FP, similar to the in vitro IEC/PBMC model, increased galectin-4 or galectin-3 over galectin-9 ratios were observed in the murine influenza vaccination model. This was associated with improved vaccine immune response determined as increased DTH response. More research is needed in order to unravel possible mechanisms implicated and deciphering the bioactive components responsible for the effects observed.

## Figures and Tables

**Table 1:** Sequence of custom-made primer and corresponding accession number.

Gene ID	Accession Number	Forward Primer Sequence (5′−3′)	Reverse Primer Sequence (5′−3′)
TNFα	NM_013693.3	AACGGCATGGATCTCAAAGA–	TTTCTCCTGGTATGAGATAGCAAATC

## Data Availability

Data is contained within the article or Supplementary Materials.

## Supplementary Materials

The following are available online at https://www.mdpi.com/2076-393X/9/3/254/s1, Figure S1: Flow cytometry analysis of T- and B-cell populations in spleen and MLN, Figure S2: Relative mRNA expression in colon, Figure S3: Ratio mRNA expression and serum galectins. Figure S4: Ratio galectin-4/galectin-3 mRNA in ileum and correlations of RORγ/TGFβ3 and serum galectins.
