# Supplementary material for: A Fermented Milk Matrix Containing Postbiotics Supports Th1- and Th17-Type Immunity In Vitro and Modulates the Influenza-Specific Vaccination Response In Vivo in Association with Altered Serum Galectin Ratios
Source: Vaccines (Basel). 2021 Mar 13;9(3):254. doi: 10.3390/vaccines9030254 (PMC7998404; doi:10.3390/vaccines9030254)
Supplement: Supplementary file 1 [file vaccines-09-00254-s001.pdf]

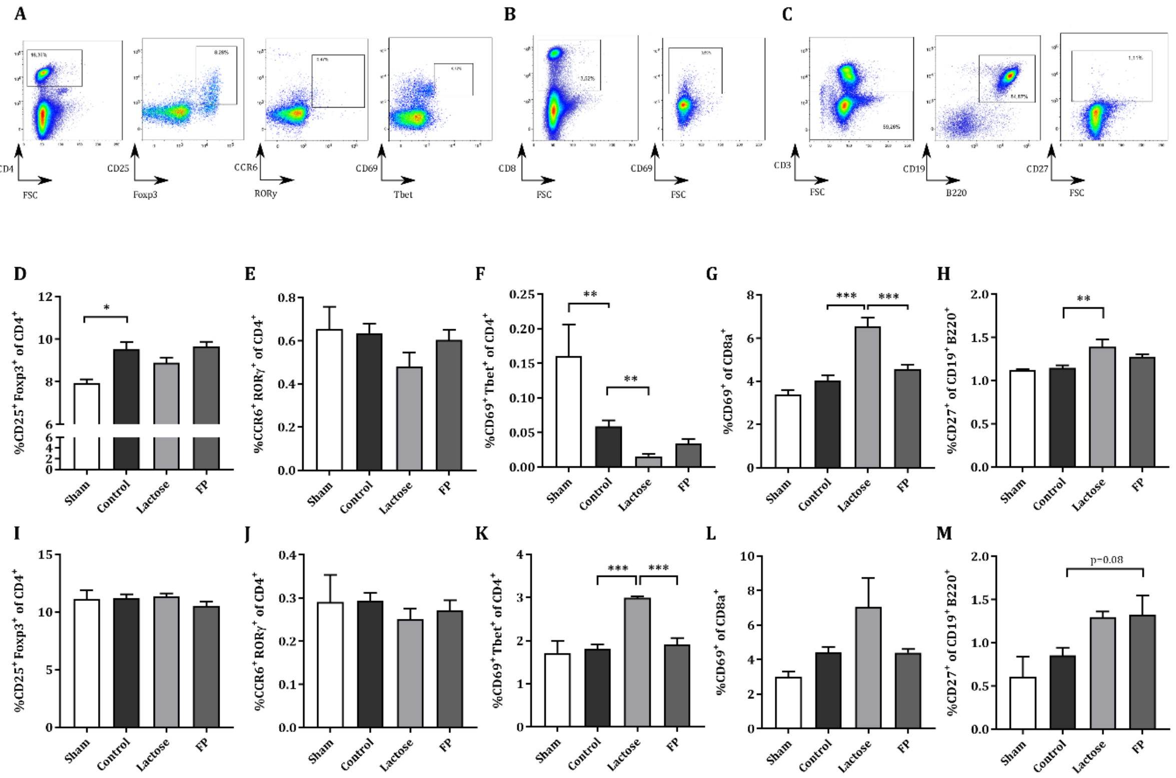

Figure S1. Flow cytometry analysis of T- and B-cell populations in spleen and MLN. On day 31, the spleens and MLNs were collected and cell suspensions were obtained. Representative $CD4^+$ T-cell (**A**), $CD8^+$ T-cell (**B**) and B-cell (**C**) plots are shown. Percentages of regulatory T-cells (**D**), Th17 (**E**), Th1 (**F**), activated $CD8^+$ T-cells (**G**) were determined in the CD4+ or CD8+ lymphocyte populations in the spleen. The percentage of $CD19^+B220^+$ B-cells (**H**) in the spleen is also shown. Respectively, T- and B-cell populations for MLN are shown (**I-M**). Data are represented as mean ± SEM of sham (n = 3) and vaccinated mice (n = 9). Significant differences are show as * $p < 0.05$, ** $p < 0.01$, *** $p < 0.001$.

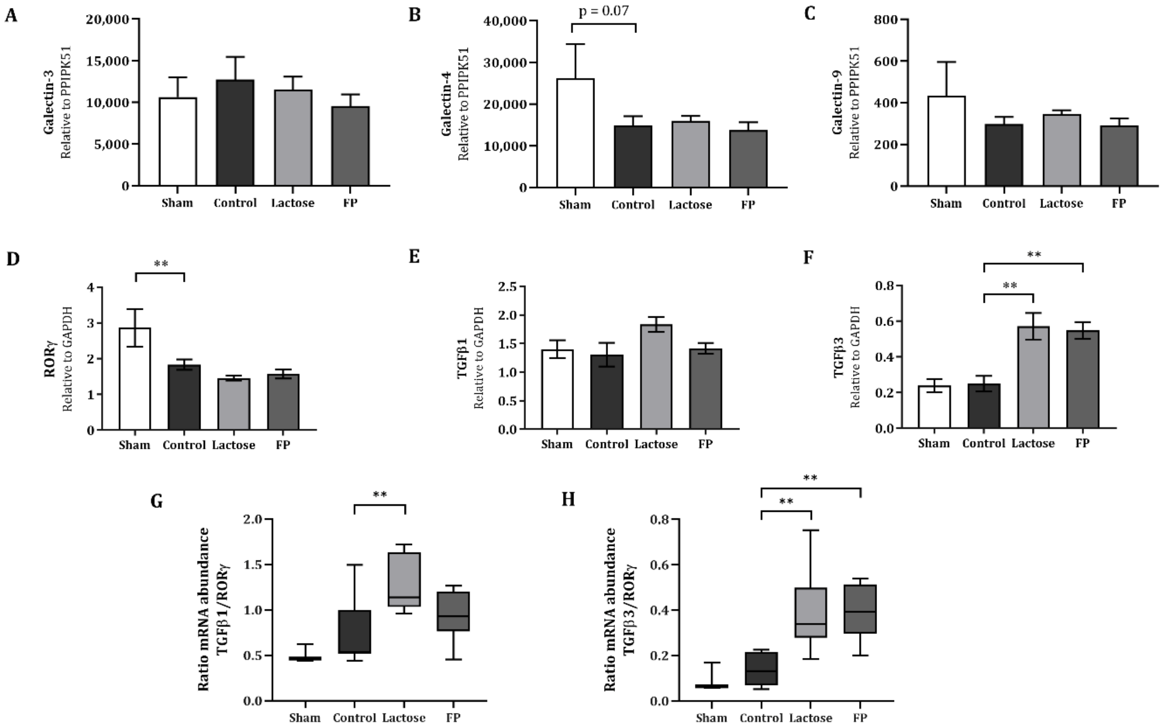

Figure S2. Relative mRNA expression in the colon. Relative mRNA expression of galectin-3 (**A**), -4 (**B**), -9 (**C**), RORγ (**D**), TGFβ1 (**E**) and TGFβ3 (**F**) were measured in the colon using RT-qPCR. TGFβ1/RORγ ratio (**G**) and TGFβ3/RORγ ratio (**H**) were calculated to represent regulatory/Th17 balance in the colon. Data are represented as mean ± SEM of sham (n = 3) and vaccinated mice (n = 9). Significant differences are show as ** $p < 0.01$.

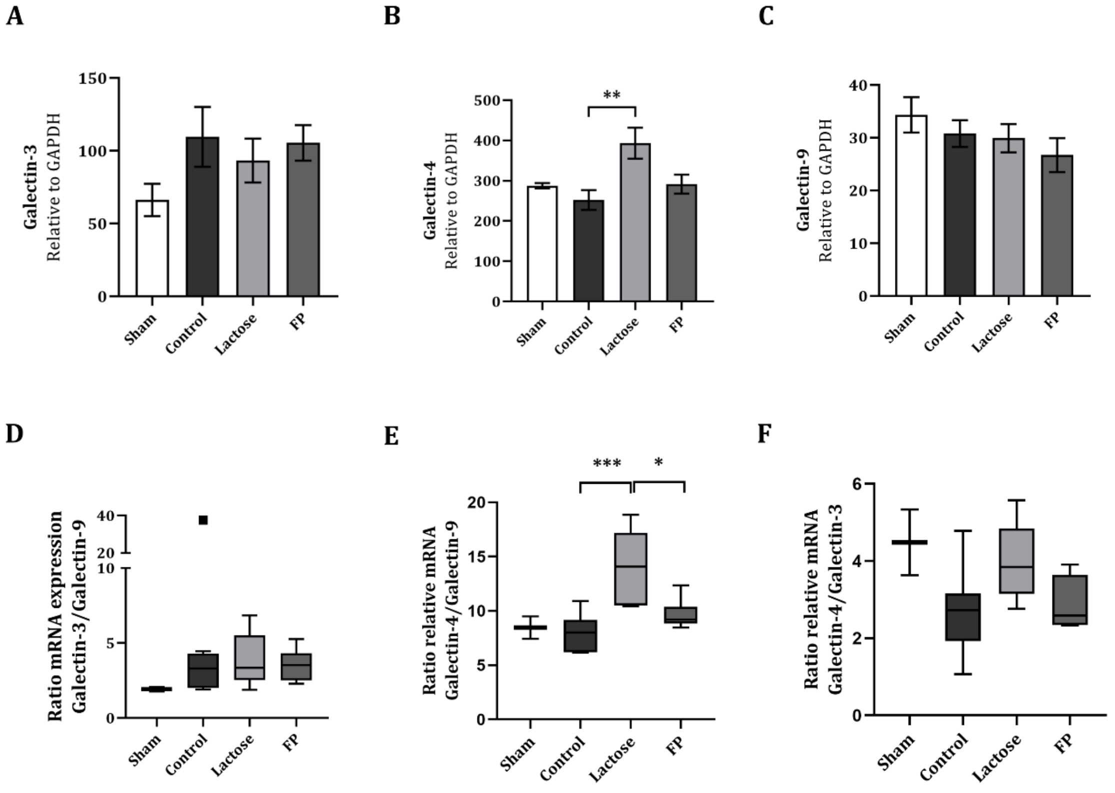

Figure S3. Ratio mRNA expression and serum galectins. Relative mRNA expression of galectin-3 (**A**), -4 (**B**), -9 (**C**), RORγ (**D**), TGFβ1 (**E**) and TGFβ3 (**F**) were measured in the ileum using RT-qPCR. TGFβ/RORγ ratios were calculated to represent regulatory/Th17 balance in the colon. Data are represented as mean ± SEM of sham (*n* = 3) and vaccinated mice (*n* = 9). Significant differences are show as * $p < 0.05$, ** $p < 0.01$, *** $p < 0.001$.

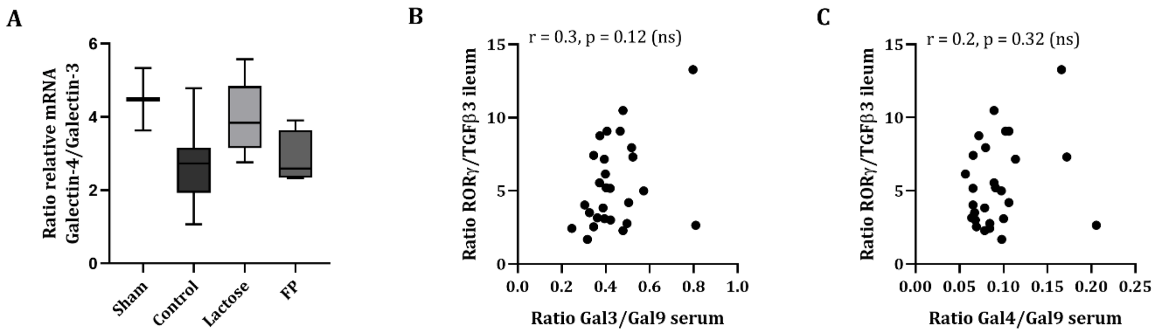

Figure S4. Ratio galectin-4/galectin-3 mRNA in ileum and correlations of RORγ/TGFβ3 and serum galectins. The ratio of galectin-4/galectin-3 mRNA expression (**A**) is shown. Additionally, RORγ/TGFβ3 ratio in ileum were correlated to serum galectin3/galectin-9 ratio (**B**) and to serum galectin-4/galectin-9 ratio (**C**) using Spearman correlation. Data are represented as mean ± SEM of sham (n = 3) and vaccinated mice (n = 9).
